# Measuring positive mental health and flourishing in Denmark: validation of the mental health continuum-short form (MHC-SF) and cross-cultural comparison across three countries

**DOI:** 10.1186/s12955-020-01546-2

**Published:** 2020-09-04

**Authors:** Ziggi Ivan Santini, Manuel Torres-Sahli, Carsten Hinrichsen, Charlotte Meilstrup, Katrine R. Madsen, Signe Boe Rayce, Melissa M. Baker, Margreet Ten Have, Marijke Schotanus-Dijkstra, Vibeke Koushede

**Affiliations:** 1 The Danish National Institute of Public Health, University of Southern Denmark, Studiestraede 6, 1455 Copenhagen, Denmark; 2 School of Social Sciences, Loughborough University, Loughborough, Leicestershire, LE11 3TU UK; 3 Vive - The Danish Center for Social Science Research, Herluf Trolles Gade 11, 1052 Copenhagen, Denmark; 4 Public Health Agency of Canada, Ottawa, Ontario Canada; 5 The Netherlands Institute of Mental Health and Addiction (Trimbos Institute), Utrecht, the Netherlands; 6 Department of Psychology, Centre for eHealth and Well-being Research, Health and Technology, University of Twente, Enschede, AE, 7500 The Netherlands; 7 Department of Psychology, University of Copenhagen, Øster Farimagsgade 2A, 1353 Copenhagen, Denmark

**Keywords:** Mental health, Positive psychology, Public health, Epidemiologic measurements, Psychometrics

## Abstract

**Background:**

The Mental Health Continuum–Short Form (MHC-SF) is a measure of positive mental health and flourishing, which is widely used in several countries but has not yet been validated in Denmark. This study aimed to examine its qualitative and quantitative properties in a Danish population sample and compare scores with Canada and the Netherlands.

**Methods:**

Three thousand five hundred eight participants aged 16–95 filled out an electronic survey. Both the unidimensional and multidimensional aspects of the Danish MHC-SF were studied through bifactor modelling. Cognitive interviews examined face validity and usability.

**Results:**

The general score of the Danish MHC-SF was reliable for computing unit-weighted composite scores, as well as using a bifactor model to compute general factor scores or measurement models in an SEM context. Nonetheless, subscale scores were unreliable, explaining very low variance beyond that explained by the general factor. The participants of the qualitative interviews observed problems with wording and content of the items, especially from the social subscale. The general score correlated with other scales as expected. We found substantial variation in flourishing prevalence rates between the three cultural settings.

**Conclusions:**

The Danish MHC-SF produced reliable general scores of well-being. Most of the issues observed regarding the subscale scores have been shown in previous research in other contexts. The further analysis of indices of the bifactor model and the inclusion of qualitative interviews allowed for a better understanding of the possible sources of problems with the questionnaire’s subscales. The use of subscales, the substantive understanding of the general score, as well as the operationalization of the state of flourishing, require further study.

## Introduction

The World Health Organization has defined mental health as “a state of well-being in which the individual realizes his or her own abilities, can cope with the normal stresses of life, can work productively and fruitfully, and is able to make a contribution to his or her community” [1]. The definition builds on two longstanding philosophies in well-being research and positive psychology: The concept of hedonic well-being which is based on positive emotional states like happiness, and the concept of eudaimonic well-being which focuses on positive functioning in the individual and on social experience and functioning [2].

The Mental Health Continuum (MHC) is a measure of positive mental health and flourishing, which encompasses both hedonic and eudaimonic well-being [3]. The MHC includes three dimensions of positive mental health: the emotional (hedonic), the social (eudaimonic), and the psychological (eudaimonic). Emotional well-being is based on Bradburn’s affect balance scale and overall life satisfaction from Cantril’s self-anchoring scale [4]. The emotional dimension thus covers the presence of positive affect and satisfaction with life. Social well-being is based on Keyes’ model [5], which includes both social functioning and connection to broader society. Finally, psychological well-being is based on Ryff’s model [6] and covers intrapersonal and interpersonal functioning.

Flourishing refers to a combination of high scores on both hedonic and eudaimonic well-being [3, 7, 8]. It is the maximization of these, and is therefore located on the top end of a well-being spectrum [9]. The ability to measure mental health positively in terms of flourishing has allowed investigations of the two continua model in which mental illness and mental health belong to correlated but separate dimensions. Several studies of youth and adult samples in various cultures support the two continua model. They have shown, for example, that flourishing protects against various negative outcomes in people with and without mental disorders, and that the absence of flourishing is sometimes as problematic as the presence of mental disorders, especially depression [10]. In regards to the operationalization of flourishing, while there is considerable agreement between different models of flourishing [11], Keyes’ model has a greater emphasis on social well-being as compared to other models. According to Keyes [5], and in line with the WHO definition of mental health, functioning well in life does not only pertain to emotional and psychological well-being; an individual must also function well in their respective communities and broader society.

The Mental Health Continuum - Short Form (MHC-SF) is a shorter 14-item self-administered version of the questionnaire. Since its development, the MHC-SF has been translated and validated in many different cultural contexts, for example, in Canada [12], the Netherlands [13], and other Western and Eastern countries. The MHC-SF scores can be used either as a continuous measure for well-being or to categorize mental health into three different states: flourishing, moderate, and languishing mental health. To be flourishing in life, individuals must exhibit high levels (upper tertile of the possible scores) on both the hedonic and eudaimonic well-being dimensions; in contrast, a languishing individual exhibits low levels (lower tertile of the possible scores) on both the hedonic and eudaimonic well-being dimensions [3]. Individuals not meeting criteria for either flourishing or languishing are considered to be moderately mentally healthy. Thus, the categorization parallels the scheme employed to diagnose major depression disorder wherein individuals must exhibit just over half of the total symptoms. According to Keyes’ theoretical model [3] and empirical studies [10], all three categories (flourishing, languishing, moderate) can occur in the presence or absence of mental illness. The operationalization of the MHC-SF into categories has received substantial interest in research because it provides a theoretically-driven cut-point (as opposed to data-driven) for different levels of mental health, and can be used more practically in epidemiological investigations into risk and protective factors of various outcomes. For example, according to a nationally-representative study of the Dutch population, flourishing, as compared to non-flourishing, reduced the risk of first and recurrent incidence of mood disorders by 28% and anxiety disorders by 53% over a three-year period [14].

**Fig. 1:**
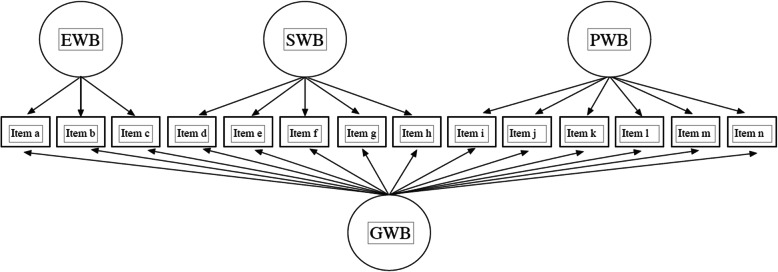
MHC-SF Bi-factor model

While validation studies have commonly supported the original three-factor structure, recent studies have questioned the goodness-of-fit of this model. They propose an alternative bifactor model (see Fig. 1) that offer a superior explanation of the scale’s inner structure [15–17]. Notwithstanding, the substantive interpretation of some indices of such bifactor models could be developed further, as it has been done for other measures of well-being or quality of life [18, 19]. This has implied an underutilization of the potentials of bifactor modelling which allows for (a) studying the partitioning of variance when an instrument assesses both general and domain-specific sources of variance, (b) contrasting if the measure is “essentially unidimensional” but with nuisance dimensions, (c) judging whether multidimensional item response data have a strong enough general factor to justify a unidimensional measurement model, and (d) determining the adequacy of a total score and what, if anything, one might gain by scoring subscales [18, 19]. These questions are pertinent to the MHC-SF since its scores are intended to measure both general and domain-specific measures.

The MHC-SF may be an appropriate scale for measuring positive mental health and flourishing in Danish population studies, and no study has validated the MHC-SF in a Danish context. Further, to our knowledge, the MHC-SF has not previously been validated qualitatively in any setting. Tackling this gap in the current understanding of the MHC-SF could complement and improve validation research for the scale, as psychometric testing alone is not sufficient to develop valid questionnaires [20]. It is important to assess questionnaires qualitatively, given that their limitations may not be evident until people are asked about their experience of filling them out. Thus, this study aims to validate the MHC-SF both psychometrically (factor structure) and qualitatively by assessing face validity and usability through cognitive interviews among a large Danish population sample. Further, since the MHC-SF is used widely internationally, it may be informative to explore how flourishing rates vary between different countries based on the MHC-SF flourishing operationalization, which may also serve as an indicator of how valid the cut-off scores are. Two other countries (to our knowledge) had access to nationally representative data that included the MHC-SF, which was also measured around the same time as the Danish survey (2016): Canada (2015) and the Netherlands (2013–15). Therefore, an additional aim is to compare the prevalence of flourishing in Denmark with scores representative of Canada and the Netherlands. Due to the scarcity of literature regarding differences between countries, we did not make any hypothesis regarding the cross-cultural comparison.

## Methods

### Study design

Our primary sample consisted of data from a national cross-sectional survey *The Danish Mental Health and Well-being Survey 2016* [21]. The survey was carried out by Statistics Denmark. A random representative sample of Danish men and women aged 16 years and above was drawn from the Danish Civil Registration System. Statistics Denmark sent an electronic letter to the sampled individuals in October 2016 with information about the study and an invitation to participate. After a week, a reminder letter was sent, and after yet another week, a final reminder was sent. More information about the data methodology and sample can be found elsewhere [21]. Apart from our primary sample, we also used an additional sample for cognitive interviews (see the section on Face validity and usability), and samples from Canada and the Netherlands for the cross-cultural comparison (see Appendix).

### Sampling

In total 10,250 individuals (5050 men and 5200 women) were contacted. Apart from the target group in terms of age (16 years old or above), there were no specific inclusion or exclusion criteria. Invited individuals could choose not to participate, and those choosing not to participate were given the option to provide information as to why they chose not to. In terms of non-response, 5854 did not respond to the invitation to participate, 463 only partially completed the survey, 183 refused to participate, three could not participate due to language barriers, 213 could not participate due to privacy protection, 26 could not participate due to medical conditions or disability, and three could not participate due to language barriers. Thus, out of the invited 10,250 individuals, a total of 3508 individuals (1656 men and 1852 women) participated in the web-based survey, resulting in a response rate of 34%.

### Ethics

There is no formal agency for ethical approval of questionnaire-based survey studies in Denmark. The study complies with the Helsinki 2 declaration on ethics and is registered with the Danish Data Protection Authority. The application of the survey met confidentiality and privacy requirements. The respondents’ voluntary completion and returning of the survey questionnaires implied consent.

### Measures

All measures included in this study were self-administered.

#### Mental health continuum-short form (MHC-SF)

The 14-item MHC-SF measures positive mental health during the past month with one hedonic dimension corresponding to emotional well-being and two eudaimonic dimensions corresponding to social and psychological well-being (see Table 1). The items are all positively worded. All items are asked using the following format: “During the past month, how often did you feel …? ”. Response categories are coded as ‘never’ (0), ‘once or twice’ (1), ‘about once a week’ (2), ‘about two or three times a week’ (3), ‘almost every day’ (4), ‘every day’ (5). Item f was originally worded “society is becoming a better place for people like me”, but following Keyes’ [22] recommendation to change the wording of the item, the Danish version instead includes the revised item “society is becoming a better place for all people”.

**Table 1:** Items included in the Mental Health Continuum-Short Form (MHC-SF) questionnaire^a^

Theoretical dimension	In the past month, how often did you feel … I løbet af den sidste måned, hvor ofte har du følt …
**Emotional well-being**	
− Happiness	a) Happy Dig glad
− Intererest	b) Interested in life Dig interesseret i livet
− Life satisfaction	c) Satisfied Dig tilfreds med livet
**Social well-being**	
− Social contribution	d) That you had something important to contribute to society At du havde noget vigtigt at bidrage med til samfundet
− Social integration	e) That you belonged to a community (like a social group, your neighborhood, your city) At du hørte til i et fællesskab (fx en gruppe eller dit nabolag)
− Social actualization	f) That our society is a good place, or is becoming a better place, for all people At vores samfund er et godt sted, eller er ved at blive et bedre sted, for alle mennesker
− Social acceptance	g) That people are basically good At mennesker generelt er gode
− Social coherence	h) That the way our society works makes sense to you At den made vores samfund fungerer på giver mening for dig
**Psychological well-being**	
− Self-acceptance	i) That you liked most parts of your personality At du kunne lide de fleste sider at din personlighed
− Mastery	j) Good at managing the responsibilities of your daily life At du var. god til at håndtere forpligtelserne i din hverdag
− Positive relations	k) That you had warm and trusting relationships with others At du havde varme og tillidsfulde relationer til andre
− Personal growth	l) That you have experiences that challenge you to grow and become a better person At du havde oplevelser, der udfordrede dig til at vokse som menneske
− Autonomy	m) Confident to think or express your own ideas and opinions Dig sikker i at tænke eller udtrykke egne ideer eller holdninger
− Purpose in life	n) That your life has a sense of direction and meaning to it At dit liv har en form for retning eller føles meningsfuldt

^a^Answers options were: “never (aldrig)”, “once or twice a month (én eller to gange om måneden)”, “about once a week (ca. én gang om ugen)”, “two or three times a week (ca. to eller tre gange om ugen)”, “almost every day (næsten hver dag)”, “every day (hver dag)”

The continuous total on the MHC-SF is calculated by summing the scores of each item, which results in a total score ranging from 0 to 70. Thus, a higher score indicates a higher level of positive mental health. In this study’s validation analyses, we primarily used the continuous total MHC-SF score when relevant (convergent validity, discriminant validity, content validity). In the cross-cultural analysis, we used both the continuous score as well as its categorical operationalization.

Categories for positive mental health were generated according to Keyes’ criteria [22]. Individuals who scored 4 (‘almost every day’) or 5 (‘every day’) on at least one item that measured emotional well-being, and also scored 4 or 5 on at least six of the eleven items of the combined scale of social and psychological well-being were categorized as ‘flourishing’. Individuals who scored 0 (‘never’) or 1 (‘once or twice’) on at least one item of emotional well-being and also scored 0 or 1 on at least six of the eleven items of the combined scale of social and psychological well-being were categorized as ‘languishing’. Individuals who were neither ‘flourishing’ nor ‘languishing’ were categorized as having ‘moderate’ mental health.

Before the initiation of the survey, the MHC-SF was translated into Danish through forward-translation and back-translation. The details of the translation methodology have been described by Sousa and Rojjanasrirat [23], and it has been applied successfully in translations of mental health and well-being measures in the Scandinavian setting [24, 25].

### Other measures

We included five additional measures in the validation study to assess relations of the MHC-SF with similar variables and other concepts expected to be associated with well-being:

#### Who-5

Covers overall well-being, five items which are scored from 0 to 5, then summed and multiplied by 4, and scored into a continuous scale from 0 to 100. High scores indicate high levels of well-being [26].

#### Self-rated health (SRH)

A single item for self-rated health which asks respondents to rate their overall health (physical as well as mental), five response options which range from poor to excellent (1–5). Higher scores indicate better self-rated health [27].

#### Discomfort and pain

Six items which measure symptoms of discomfort and pain within the past 2 weeks; Shoulder or neck; Back or lower back; Arms, hands, legs, knees, hips or joints; Headache; Stomach-ache; Difficulties sleeping. Each item is coded 0 = symptom not present, 1 = symptom present. Items are summed to score on a scale ranging from 0 to 6, with higher scores indicating a higher number of symptoms [27].

#### The perceived stress scale (PSS) [28] covering perceived stress and coping

Ten items that are each scored from 0 to 4. Positive items are reversed and summed into a scale ranging from 0 to 40. Higher scores indicate higher levels of perceived stress.

#### The patient health questionnaire for depression and anxiety (PHQ-4)

Data on poor mental health was collected using the PHQ-4 which asks respondents about their experience of core depressive and anxiety symptoms over the past 2 weeks as specified by DSM-IV [29]. There are four items for depression/anxiety; each item is given a score from 0 to 3 and then scored into a continuous scale ranging from 0 to 12 Higher scores indicate a high level of depression/anxiety.

Other variables included in the present study were: sex (male, female), age, education (primary or unknown, youth education, short-cycle higher education (2–2½ years of full-time study), medium-cycle higher education (3½-4 years of full-time study), long-cycle higher education (5–6 years of full-time study), employment status (employed, not employed or unknown), and living arrangements (single, married or with a partner).

### Steps of validation and statistical procedures

Validation of the MHC-SF scale examined: 1) factor structure assessing content validity, goodness-of-fit and measurement invariance through confirmatory factor analysis, as well as internal consistency and relations to other or similar measures, and 2) face validity and usability. Psychometric analyses were completed using Stata, the R statistical language and programming environment [30], and the *lavaan* package for confirmatory factor analysis in R [31]. Apart from the validation analyses, we also performed an additional cross-cultural comparison of the continuous and categorical MHC-SF estimates in Denmark with Canada and the Netherlands.

#### Factor structure

We examined total scores for floor and ceiling effects. Instruments exhibit floor or ceiling effects if more than 15% of respondents record the lowest or highest score [32].

We conducted confirmatory factor analysis (CFA) over two randomly created subsets to analyze global goodness-of-fit (*n* = 694) and measurement invariance (*n = 2814)*. We used an unweighted least squares estimator with means and variance adjusted (ULSMV). We modelled three different structures: two first-order models with one and three correlated factors respectively, and a hierarchical or bifactor model with three domain-specific factors. In the three-factor model, items a–c loaded on the latent variable of emotional well-being, items d–h on social well-being, and items i–n on psychological well-being [33]. In the bifactor model (see Fig. 1), every item loaded onto one of the three domain-specific factors — as specified in the three-factor model — and also on a general well-being factor [17].

As recommended by Hoyle and Panter [34], we used several fit indices including the Root Mean Square Error of Approximation (RMSEA), the standardized root mean square residual (SRMR), the Comparative Fit Index (CFI), and the Tucker-Lewis Index (TLI). Values greater than 0.95 for the CFI and TLI were considered to reflect good model fit. RMSEA and SRMR values of 0.06 or less were considered to indicate good fit, although values up to .08 were considered acceptable [35]. We evaluated measurement invariance across sex (women vs men), age groups (16–54 years of age vs 55+), and education (primary or unknown vs youth education vs short-long cycle educations), examining differences in Alternative Fit Indexes. We considered a model invariant when the respective constraint produced at most −.01 change in CFI, paired with changes of up to .015 in RMSEA, and .030 (for metric invariance) or .015 (for scalar or residual invariance) in SRMR [36].

Besides the general fit of the models to the data, we analyzed further the bifactor model to study the potential multidimensional and unidimensional uses of the MHC-SF scores implied in the three-factor and the one-factor solutions. The two alternatives have been considered plausible for the conceptualization of well-being, and several recent validations of MHC-SF versions have consistently included at least two of the three models [16, 17]. Following Rodriguez, Reise and Haviland [37], several aspects of the (Danish) MHC-SF were evaluated through a bifactor lens: (1) the reliability of unit-weighted composite scores; (2) the use of a set of items to compute factor scores or to identify a latent variable in an SEM context; and (3) whether multidimensional (bifactor) data are “unidimensional enough” to specify a unidimensional measurement model in an SEM context. For the first aspect, we calculated omega (ω), omega hierarchical (ω_hs_), and their ratio (ω_hs_/ω). For the second, we estimated indices of factor determinacy (FD, benchmark >.90) and construct reliability or replicability (*H*, benchmark: >.70). For the third aspect of the evaluation, we analysed the explained common variance by the general factor on all items (ECV) and on each individual item (I-ECV), the percentage of uncontaminated correlations (PUC), and the relative parameter bias as the difference between an item’s loading in the unidimensional solution and its general factor loading in the bifactor, divided by the general factor loading in the bifactor.

We assessed convergent validity by calculating correlations between the MHC-SF continuous score and WHO-5, and discriminant validity by calculating correlations between the MHC-SF and SRH, education, symptoms of discomfort and pain, PSS, and PHQ-4. We hypothesized that the MHC-SF scores would show a strong positive correlation with well-being (WHO-5) [38], moderate positive association with SRH, and moderate negative associations with scales measuring the negative aspects of physical or mental health status (symptoms of discomfort and pain, PSS and PHQ-4) [12, 14] (based on Cohen’s rule of thumb, i.e. small: r = 0.1; moderate = 0.3; large = 0.5 [39]).

Based on the findings of recent Danish health and morbidity studies, we hypothesized that the scale would show a positive association with education [27, 40]. The association was hypothesized to be weak to moderate based on recent studies suggesting that well-being is less sensitive to socioeconomic patterns compared to poor mental health [41]. Differences in scores across sex and education were assessed using linear regression analysis.

#### Face validity and usability

Cognitive interviewing techniques were used to examine the face validity of the scale (i.e., do people understand the questions in the way they were intended) and usability (i.e., how participants process and respond to the scale). Eleven face-to-face interviews, all in Danish, were conducted with six men and five women age range 20–77 years. Participants were selected with the aim to have variation in age, sex and education, which are attributes known to be associated with mental health and health literacy [42]. The interviews followed an interview protocol that was developed in accordance with the recommendations by Gray [43]. All interview questions were non-leading, non-directing and neutrally framed (e.g. “how did you experience responding to the questionnaire?”). During the interview, prior scripted and spontaneous open-ended probes were used. All interviews were recorded and summarized in writing using a literary style [44]. The software program QSR NVivo 11 was used to assist managing and analyzing the qualitative data; allowing to work with the written summaries and the audio files simultaneously. The interview data were analyzed using the Framework approach [45]. The applied framework consisted of six a priori themes based on the Tourangeau et al. model for survey response [46]: *comprehension (overall)*, *comprehension (item-specific)*, *retrieval*, *judgement*, *response*, and *other* (relevant passages, not fitting with other themes). Following a thorough familiarization with the data, the data were categorized according to the six themes. The content of each theme was compared across participants with the aim of identifying potential challenges in the response process and different ways of interpreting and answering the questions. Next, patterns and links between themes were identified to explain why problems occurred. Preliminary findings were discussed within the research team. A summary of the findings is presented in the results section structured according to the themes used in the analysis (the themes *retrieval* and *other* are not reported as there were no notable results pertaining to them). The findings are supported by illustrative quotes.

#### Cross-cultural comparison of MHC-SF scores and flourishing prevalence rates

Total MHC-SF scores and categories for well-being were computed with weights applied to generate nationally representative estimates using the Stata svy command. The Danish scores were reported along with scores based on data representative of two other countries, specifically Canada and the Netherlands. The Dutch scores were, however, based on a version with revised response categories (see Appendix) to make it easier for the respondents to recall. These revised response categories were: never (1), rarely (2), sometimes (3), regularly (4), often (5), or (almost) always (6). The total MHC-SF scores and categories for well-being were reported for each country as well as stratified by age and sex. Information regarding survey and sampling in Canada and the Netherlands is provided in the Appendix.

## Results

### Respondent characteristics

From a total of 3508 respondents, 1852 (52.8%) were women. With a mean age of 52.1, 319 (9.1%) were aged 16–25, 735 (21.0%) were 26–44 years old, 1437 (41.0%) were aged 45–64, and 1017 (28.9%) were 65–95. Among respondents, 2528 (72.0%) were either married or living with a partner; 1919 (54.7%) were employed; and 1220 (34.8%) were educated beyond youth education (Further details in Table 2.)

**Table 2:** Characteristics of the study sample

Characteristic	Category	*n*	%	Response rate (%)^a^
Total number of respondents (N)		3508	100	34
Sex	Female	1852	52.8	36
	Male	1656	47.2	33
Age (years)	16–25	319	9.1	20
	26–34	282	8	21
	35–44	453	12.9	28
	45–54	667	19	38
	55–64	770	21.9	50
	65+	1017	29	43
Education	Primary or unknown	831	23.7	24
	Youth education	1457	41.5	36
	Short-cycle higher education	170	4.8	42
	Medium-cycle higher education	627	17.9	47
	Long cycle higher education	423	12.1	44
Employment status	Employed	1919	54.7	38
	Not employed or unknown	1589	45.3	31
Living arrangements	Single	980	28	26
	Married or with partner	2528	72	39

^a^Response rate in relation to the invited survey sample

### Factor structure

The data presented good sampling adequacy (Kaiser-Meyer-Olkin’s MSA = .93; Bartlett’s sphericity test: χ^2^ = 6806.09, *df* = 91, *p* < .001). Multivariate normality tests indicated that the scores were not normally distributed (Henze-Zirkler = 60, p < .001; Royston = 117, p < .001). This seemed to be due to skewness rather than kurtosis issues (Mardia’s test: skewness = 660.4, kurtosis = − 0.86), which is consistent with what was observed histograms, in which the MHC-SF total scores appeared to be skewed left. Although neither floor nor ceiling effects were observed for the overall continuous score, eight items (a, b, c, e, j, k, m, n) showed ceiling effects (26–43% of responses in the highest level [32];). To compensate for both nonnormality and censored variables, we used a robust estimator with mean and variance adjusted (ULSMV).

According to global goodness-of-fit indices (Table 3), MHC-SF one-factor (χ^2^ (76) = 354, CFI = .97; TLI = .97; SRMR = .061; RMSEA = .073) and three-factor (χ^2^ (73) = 266, CFI = .98; TLI = .98; SRMR = .050; RMSEA = .062) models presented acceptable fit, with RMSEA over the ideal cutoff point. The bifactor model presented excellent model fit (χ^2^ (62) = 122, CFI = 0.99; TLI = 0.99; SRMR = .030; RMSEA = 0.037) and was therefore the selected model used in subsequent analyses of measurement invariance and internal consistency. Although the bifactor structure presented the best fitting, given that neither solution presented unacceptable global fit indices, we explored further the bifactor structure to analyze both the unidimensional and multidimensional aspects of the Danish MHC-SF.

**Table 3:** Goodness-of-fit indices based on confirmatory factor analysis

	SBχ^2^	*df*	χ^2^/*df*	CFI	TLI	SRMR	RMSEA [90%CI]
One-factor	354	76	4.7	.97	.97	.061	.073 [.065, .080]
Three-factor	266	73	3.6	.98	.98	.050	.062 [.054, .070]
Bifactor	122	62	2	.99	.99	.030	.037 [.028, .047]

Note: *MHC-SF* Mental Health Continuum – Short Form, *SBχ*^*2*^ Satorra-Bentler scaled chi-square, *df* Degrees of freedom, *CFI* Comparative fit index, *TLI* Tucker-Lewis index, *RMSEA* Root mean square error of approximation, *SRMR* Standardized root-mean-square residual

In the bifactor model (see Table 4), item loadings onto the general factor were all large (.61–.86). The Emotional factor presented even, moderately sized domain-specific loadings (.38–.46). On the other hand, both the Social and the Psychological factors presented two items not significantly different from zero (*p* > .05; items d, e, l, and n). Except for the loadings of items f and h in the Social subscale, items of the Danish MHC loaded noticeably more on the general factor than on their domain-specific factor. This is consistent with the common variance of each item explained by the general factor (I-ECV) for those two items, which were 49 and 50% respectively. For all the other items, the common variance was mostly explained by the general factor (I-ECV = .71–1.00).

**Table 4:** Items, factor loadings and statistical indices for the bifactor model of the Danish MHC-SF scores

	General	Emotional	Social	Psychological	I-ECV	RPB
***Bifactor Model loadings***						
Item a	.73*** (.02)	.46*** (.03)			0.71	4.1%
Item b	.76*** (.02)	.38*** (.03)			0.81	3.2%
Item c	.82*** (.02)	.41*** (.03)			0.82	3.0%
Item d	.71*** (.02)		−.05 (.03)		1.00	3.4%
Item e	.69*** (.02)		.06° (.03)		0.99	1.0%
Item f	.60*** (.03)		.62*** (.04)		0.49	7.6%
Item g	.62*** (.03)		.34*** (.03)		0.77	4.6%
Item h	.61*** (.03)		.61*** (.04)		0.50	7.5%
Item i	.74*** (.02)			.28*** (.05)	0.85	2.1%
Item j	.69*** (.03)			.41*** (.06)	0.75	3.5%
Item k	.78*** (.02)			.17*** (.04)	0.96	0.3%
Item l	.73*** (.02)			.02 (.05)	1.00	1.8%
Item m	.67*** (.02)			.32*** (.06)	0.84	2.6%
Item n	.86*** (.02)			.03 (.04)	1.00	1.7%
***Statistical Indices for evaluating bifactor model***						
Omega (ω)	.91	.87	.79	.87		
Omega hierarchical for general factor (ω_h_) and subscales (ω_s_)	.88	.18	.17	.05		
ω_hs_/ω	96.7%	20.7%	21.5%	5.7%		
Construct Replicability (*H*)	.94	.39	.58	.30		
Factor Determinacy (FD)	.96	.76	.85	.63		
Explained Common Variance (ECV)	80.0%					
Percentage of Uncontaminated Correlations (PUC)	69%					

*Notes.* I-ECV = item explained common variance, i.e. percent of common variance due to the general factor. RBP = *relative parameter bias* as the difference between an item’s loading in the unidimensional solution and its general factor loading in the bifactor (i.e., the truer model), divided by the general factor loading in the bifactor. * *p* ≤ 0.05, ** *p* ≤ 0.01, *** *p* ≤ 0.001

The unit-weighted scores for both the complete set of items and the subscales presented a high percentage of reliable common variance (ω = .79–.91). Nonetheless, when considering the hierarchical structure, the emotional (ω_s_ = .18), social (ω_s_ = .17), and psychological (ω_s_ = .05) subscales presented low proportion of reliable variance above and beyond that explained by the general factor (ω_h_ = .88). The general factor explained an extremely high proportion of the total reliable common variance of the entire set of items (.88/.91 = 96.7%). In contrast, the Emotional (.18/.87 = 20.7%) and Social (.17/.79 = 21.5%) factors explain no more than a quarter of the reliable common variance of their subsets of items beyond that explained by the general factor. The Psychological factor explained almost no reliable common variance (.05/.87 = 5.7%) above that explained by the general factor.

The factor scores of the constructs followed a pattern similar to that of the unit-weighted scores. While the general factor scores showed both sufficient construct replicability (*H* = .94) and factor determinacy (FD = .96), the emotional (*H* = .39, FD = .76), social (*H* = .58, FD = .85), and psychological (*H* = .30, FD = .63) factor scores offered below-acceptable levels.

Given that both the unit-weighted and factor scores showed to be reliable only for the general factor, we studied whether the multidimensional (bifactor) data was “unidimensional enough” to specify a unidimensional measurement model in an SEM context. The explained common variance by the general factor (ECV = .80) could reflect a potentially unidimensional item set, which paired with the high percentage of uncontaminated correlations (PUC = 69%) could imply very little difference in the factor loadings between a unidimensional model and the general factor in a bifactor model. To assess this, we computed the relative parameter bias as the difference between an item’s loading in the unidimensional solution and its general factor loading in the bifactor, divided by the general factor loading in the bifactor (the ‘truer’ model). We found that the average relative bias across items was very low (3.3%), with a minimum of 0.34% and a maximum of 7.6%.

Being the bifactor model the best solution to represent a general factor of well-being within multidimensional data, we studied further the measurement invariance across different groups. Measurement invariance (Table 5) was sustainable across sex, age groups, and education. For all grouping variables and levels of measurement invariance (weak against configural, strong against weak) differences in alternative fit indexes (ΔCFI, ΔTLI, ΔRMSEA, ΔSRMR) were below our cut-off points.

**Table 5:** Measurement invariance for the MHC-SF bi-factor model by sex, age, and education, estimated through differences in alternative fit indices

	χ2(df)	CFI	TLI	RMSEA	SRMR	Δχ2(Δdf)	ΔCFI	ΔTLI	ΔRMSEA	ΔSRMR	Decision
**MHC-SF (sex)**											
Configural invariance	356.5*** (166)	.996	.995	.029	.027	–	–	–	–	–	–
Metric invariance	387.5*** (190)	.996	.996	.027	.030	31** [24]	.000	.001	−.002	.003	Accept
Scalar invariance	412.4*** (204)	.995	.996	.027	.031	25** [14]	−.001	.000	.000	.001	Accept
**MHC-SF (age)**											
Configural invariance	493.9*** (166)	.992	.992	.038	.027	–	–	–	–	–	–
Metric invariance	553.9*** (190)	.992	.992	.037	.032	60*** [24]	.000	.000	−.001	.005	Accept
Scalar invariance	662.5*** (204)	.989	.990	.040	.033	109*** [14]	−.003	−.002	.003	.001	Accept
**MHC-SF (education)**											
Configural invariance	373.6*** (270)	.997	.997	.020	.028	–	–	–	–	–	–
Metric invariance	423.8*** (318)	.997	.998	.019	.032	50 (48)	.000	.001	−.001	.004	Accept
Scalar invariance	661.6*** (346)	.992	.994	.031	.034	238*** [28]	−.005	−.004	.012	.002	Accept

Note: *MHC-SF* Mental Health Continuum – Short Form. *SBχ*^*2*^ Satorra-Bentler scaled chi-square, *df* Degrees of freedom, *CFI* Comparative fit index, *TLI* Tucker-Lewis index, *RMSEA* Root mean square error of approximation, *SRMR* Standardized root-mean-square residual. *p ≤ .05; **p ≤ .01; ***p ≤ .001

In terms of convergent validity (Table 6), the MHC-SF correlated positively and more strongly with the WHO-5 than with other measures. In terms of discriminant validity, there was a strong negative correlation with the PHQ-4 and the PSS, a moderate positive correlation with SRH, and a moderate negative correlation with symptoms of discomfort and pain. Finally, there was a statistically significant but weak correlation between MHC-SF and education.

**Table 6:** Relations to other or similar measures

	ω	MHC-SF	WHO-5	Self-rated health	Education	PHQ-4	PSS	Symptoms of discomfort and pain
- MHC-SF	0.88	–						
- WHO-5	0.89	0.72*	–					
- SRH	–	0.40*	0.48*	–				
- Education	–	0.08*	0.02	0.13*	–			
- PHQ-4	0.80	−0.54*	−0.69*	−0.40*	−0.12*	–		
- PSS	0.82	−0.58*	−0.70*	−0.41*	−0.10*	0.70*	–	
- Symptoms of discomfort and pain	0.67	−0.30*	−0.43*	− 0.48*	− 0.11*	0.36*	0.41*	–

Note: MHC-SF = Mental Health Continuum – Short Form (range 0–70)

*Statistically significant (*p* < 0.05)

### Face validity and usability

As discussed further below, the qualitative study sheds light upon some of the issues identified through the bifactor modelling. This concerns especially the items pertaining to the social subscale that offered the highest unique variance over the variance shared with the general factor.

#### Comprehension (overall)

Several participants experienced difficulties completing the scale, with reactions to the questionnaire being often negative. The main problems were comprehending the questions and their relevance. The layout of the questionnaire was considered to disrupt the flow of reading because the first part of the questions (*Within the past month, how often did you feel …*) was only written once at the top, i.e. respondents felt they had to read the first part again for every new item in order to comprehend the entire item. Overall, the items in the MHC-SF scale were considered as being unusual or characterized as something people “*usually do not think about*” or do not “*consciously reflect upon*”, making them hard to answer and prolonging the decision-making process.

#### Comprehension (item-specific)

Items f (*That our society is becoming a better place for people*) and h (*That people are basically good*) evoked reactions or comments from most participants. They found the questions problematic to answer and irrelevant regarding their personal state of well-being or mental health since they considered the questions to be about personal political values and views. For example, a respondent stated,

" *This [item f] is really a sick question. Because it is very political and has nothing to do with me. Or, in some way it has." (male, 47 years)*

A participant explained that some questions are very sensitive to the context and external conditions, e.g. political events, media and social interactions. This was especially evident for items f (*That our society is becoming a better place for people*) and g (*That people are basically good*) as participants based their responses, for example, on global political events (e.g. one participant mentioned the election of a new president in the USA in 2016 and how this might impact the societal situation both globally and nationally). Some considered the decision-making process for item c (*Satisfied*) to be highly influenced by the way the question is contextualized by the respondent (e.g. comparing oneself with a homeless or a child in Africa is different from comparison with the neighbour next door). Another participant pondered over the use of the word ‘society’ in item f, and remarked that the question is more about decision-makers (e.g. politicians) than about the participant:

"*This is not so much about what I am in society, but how others are running our society or deciding how it has to be*" (female, 57 years)

The wording of item f puzzled several participants, and two informants pointed out that the question was ambiguous because it focuses on two matters within the same item: the current state of society, and the current developmental direction of that same society:

*"I can say, it is a shitty place, but it is getting better. Or, it is a good place, but it is getting worse. … Therefore, it is very ambiguous when you go into the phrasing here."* (male, 47 years)

Two respondents found item g (*That people are basically good*) not to be in accordance with their worldview and their understanding of human nature, as they did not find it possible to categorize people as being ‘good’. Several participants considered the wording in item g too broad and vague as it was not clear who and what the word ‘*people’* covers (close relations, the Danish population, or all people in the world). Item l (*That you have experiences that challenge you to grow and become a better person*) was considered complicated, which caused doubt about how it should be interpreted.

#### Judgement and response

The broad and vague wording, as described above, complicated the decision-making process for some participants. Likewise, items b (*Interested in life*) and d (*That you had something important to contribute to society*) were considered hard to answer, because participants found them to be too broad. Also, respondents found it difficult to assess how many times they had experienced a given feeling. According to the participants, this issue occurred because they construed some items as asking about values (e.g. political views) rather than feelings per se. This issue was especially evident for items f (*That our society is becoming a better place for people*), g (*That people are basically good*) and h (*That the way our society works makes sense to you*).

Participants had varying opinions about whether the number of response categories was appropriate or not. Some participants found the number of response categories to be appropriate, while others suggested that there should be more response categories. One participant remarked that five response categories would be appropriate, as this would give a middle option. The wording of the response categories, specifically in connection with item i (*That you liked most parts of your personality*), f (*That our society is becoming a better place for people*), and h (*That people are basically good*), were considered to be “clumsy” and “random” as these items were interpreted as asking more about fundamental personal values than about the frequency of experiencing a given feeling:

*" [ … ] it is hard to tell, if it is almost every day, or if it is one time a week, or if it is two to three times a week. Because it is a core value that I have, and therefore, I think, to me it must be almost every day, right?*" (female, 65 years)

This issue is related to the problems caused by the difficulty in assessing how many times a specific/given feeling had been experienced, as well as whether one can experience a personal value or attitude less than all the time. Altogether, there were problems with several aspects of the scale, i.e. the overall layout, the wording and thematic content in several items, as well as possible response categories.

### Cross-cultural comparison of MHC-SF scores and flourishing prevalence rates

Table 7 shows the MHC-SF scores in Denmark, in Canada and in the Netherlands. Mean scores for the total scale in Denmark was 50.0 (SD = 12.5). The highest overall MHC-SF scores were reported for Canada, followed by Denmark, and the lowest reported for the Netherlands. In Denmark, Canada and the Netherlands, there were no significant differences in terms of sex. However, in terms of age groups, the 65+ scored significantly higher than the other age groups in Denmark and Canada, while those aged 26–44 scored significantly higher than other age groups in the Netherlands. In terms of overall prevalence rates for flourishing, Canada had the highest prevalence (82.8%), Denmark rated second (64.5%), and the Netherlands rated last (38.6%). However, in terms of overall prevalence rates for languishing, Denmark rated first (3.9%), followed by the Netherlands (1.6%), and Canada (0.9%).

**Table 7:** Cross-cultural comparison of positive mental health scores representative of Denmark, Canada, and the Netherlands

			Mean		Prevalence (%)	
	Category	*n*	MHC-SF	Languishing	Moderate	Flourishing
Denmark 2016	Overall	3508	50.0	3.9	31.7	64.5
	Females	1852	49.9	3.9	31.9	64.2
	Males	1656	50.1	3.8	31.5	64.8
	16–25	319	48.8	5.1	35.2	59.8
	26–44	735	49.7	4.2	32.6	63.2
	45–64	1437	49.5	4.1	32.6	63.4
	65+	1017	52.1	2.1	26.5	71.5
Canada 2015	Overall	36,931	56.5	0.9	16.3	82.8
	Females	19,928	56.3	1.0	16.4	82.7
	Males	17,003	56.7	0.9	16.2	83.0
	16–25	4764	55.7	1.1	18.0	81.0
	26–44	10,948	56.4	0.8	17.2	82.0
	45–64	12,907	56.7	1.1	15.3	83.6
	65+	8312	57.1	0.8	14.3	85.0
The Netherlands 2013–15	Overall	4618	44.6	1.6	59.8	38.6
	Females	2559	44.9	1.5	58.5	40.0
	Males	2059	44.3	1.7	61.2	37.1
	16–25	81	44.2	0.0	65.1	34.9
	26–44	1391	45.3	1.3	54.2	44.5
	45–64	2325	44.1	1.8	62.3	35.8
	65+	821	44.6	2.3	65.1	32.6

Note: *MHC-SF* Mental Health Continuum – Short Form (range 0–70)

## Discussion

The primary aim of this study was to examine the validity of the Danish MHC-SF. To our knowledge, this is the first validation study of the MHC-SF in a Scandinavian setting. It is also the first to include a qualitative assessment of the instrument. The study of the factor structure focused on examining the unidimensional and multidimensional interpretations of the instrument through bifactor modelling. The results of the qualitative study focused on issues the interviewees had with both formal and content-related aspects of some items. The convergence of the Danish MHC-SF scores with other related measures was also analysed. Finally, we compared the scores of this Danish sample with MHC-SF scores of previous studies in different cultural contexts.

The fact that the unidimensional and the multidimensional models showed acceptable fit made it relevant to further explore the possibilities of using the MHC-SF scores in both ways. A bifactor analysis allowed us to do so as has been done before for other psychometric measurements [19, 47]. Similar to previous bifactor analyses of the MHC-SF [16, 17], we found a low reliable common variance in domain-specific scores beyond that explained by the general factor, and some extremely low loadings in the subscales. The general factor was the only one showing high reliability of unit-weighted composite scores. It was also the only one presenting enough factor determinacy and construct replicability for computing factor scores or identifying a latent variable in an SEM context. Also, and considering the unreliability of the subscales, we observed that the multidimensional data of the MHC-SF were “unidimensional enough” to specify a unidimensional measurement model in an SEM context. In sum, the examination of the factor structure through bifactor modelling supports the use of both general unit-weighted composite scores of the Danish MHC-SF in practical settings as well as general factor scores in measurement models.

Beyond the reliability of the general score, it is relevant to discuss the potential implications of the model for understanding the content of the general factor of well-being. The fact that the psychological subscale presented almost no reliable variance above and beyond the general factor may lead to the interpretation that the MHC-SF measures nothing more than what is covered by the psychological subscale. Nonetheless, when looking in detail to the specific items that are almost wholly explained by the general factor (I-ECV > .90), they pertain to both the social and psychological subscales. These are the items corresponding to *social contribution* (d), *social integration* (e), *positive relations* (k), *personal growth* (l), and *purpose in life* (n) – all related, in their content, to either belonging or meaning. This could be read as if the general score of the MHC-SF corresponds to what has been called eudaimonic well-being – with its social and psychological components. However, the items corresponding to what has traditionally been conceptualised as hedonic well-being are still highly explained by the general factor (I-ECV = .71–.82). The Danish MHC-SF seems to capture a general factor of well-being which incorporates both hedonic and eudaimonic aspects. Our analyses pose the question though, whether these different aspects of well-being can be reliably measured separately. The question of whether it is best understood as a unidimensional or multidimensional construct seems to be illuminated by these results.

Measurement invariance testing showed that the bifactor structure of the Danish MHC-SF was equivalent across sex, age group, and education level, which is a strength regarding the use of the scale in the Danish general public, allowing for potential comparisons across groups defined by these variables. The results from the convergent and discriminant validity tests suggest that the MHC-SF share common features with the WHO-5, and is inversely related to the PSS and PHQ-4, in line with previous findings [12, 14].

The qualitative study sheds light upon some of the issues identified through the bifactor modelling. Interviewees pointed out problems with some aspects of the scale such as layout, wording and thematic content in some items, and response categories. The items that the participants criticized the most were the items of the social subscale which offered the highest unique variance. If we take into consideration the participants’ interpretation of such items, it could be that items f–h of the social subscale are a source of variance which mixes general well-being with value or political positions. Such mixture was met with discomfort by the respondents. While the conceptualization [3] and construct validity [13] of the MHC-SF have been addressed in several publications, we were unable to find qualitative studies on the content and face validity of the scale. It is therefore uncertain whether the problems found in this study exist irrespective of which language version of the MHC-SF is being used or whether they pertain strictly to the Danish version of the MHC-SF. That said, some of the issues pointed out by the participants may shed light on psychometric problems of the MHC-SF that have been shown not only here, but in previous studies – especially regarding the social subscale. Therefore, the insights offered by the participants in this study may contribute to understanding issues that have been or could be problematic in other translations and the original instrument as well.

Since some questions were difficult to understand for the interviewed participants, a revision of the MHC-SF may be needed to assure content and face validity. In the Dutch version of the MHC-SF, the response categories were simplified (see the appendix) into more general statements about how often an item applies to them rather than the number of times (e.g. ‘regularly’ versus ‘two or three times a week’) [14]. Although vague quantifiers are open to interpretation frequency-wise [20, 48, 49], our qualitative analysis indicates that the Dutch response format might be a better solution in a Danish context.

The critical observations made by the participants during the cognitive interviews seem to clarify some of the issues observed in the factor analysis. It is relevant to observe that the general attitude of the interviewees was noticeably critical of the questionnaire, both in terms of content and form. This contrasts with psychometric analyses, in which the scores showed to be reliable for computing both factor and unit-weighted composite scores. It is possible that the use of cognitive interviewing techniques may have produced difficulties that respondents would not experience when completing the MHC-SF under other circumstances [50]. However, another possible reason for the MHC-SF performing well psychometrically may be that respondents, despite any reservations on the wording and response categories of the items, still managed to make an overall assessment of the well-being aspect in question.

The cross-cultural comparison between Denmark, Canada and the Netherlands showed substantial variation in flourishing prevalence rates between the three countries, with Canada having the highest prevalence of flourishing, Denmark second-highest, and the Netherlands having the lowest of the three. A flourishing prevalence rate of 82.8% for Canada was found in the 2015 sample, impliying an increase compared to a previous prevalence rate (76.9%) reported for Canada in 2012 [51]. These flourishing rates are, as far as we know, the highest ever reported, which has raised concerns about the functioning of the scale among the Canadian authors. One study involving a student sample in Tanzania also reported a remarkably high flourishing rate of 72.8% [52]. Studies using the MHC-SF to measure flourishing in African settings are scarce, but this number may be compared to a 20% flourishing rate reported in a South African sample [53]. In the current study, Denmark rated second of the three countries in terms of average well-being and percentage of flourishers (64.5%), designating the Netherlands as having the lowest prevalence of flourishing (38.6%) of the three countries included in this paper.

According to a report by the European Social Survey (not based on scores from the MHC or the MHC-SF), Denmark ranked higher than the Netherlands on both hedonic and eudaimonic well-being in 2012 [54]. Similarly, a previous European multi-country study that compared Huppert and So’s flourishing scale found that the prevalence of flourishers in 2006 was 41% in Denmark and 20% in the Netherlands [56]. Hone et al., (2014) [11] conducted a comparative study on different flourishing scales on a New Zealand sample and found that Keyes’ flourishing criteria were less conservative than Huppert and So, with approximately 15% more people qualifying as flourishers when using the MHC-SF. Thus, if one was to assume that there has not been a substantial development in the numbers of flourishers in Denmark and the Netherlands since 2006 (which may or may not be the case), the rates give some credibility to the Danish and Dutch estimates. A recent study estimating the 2012 prevalence of flourishing in Denmark according to Huppert and So’s scale also arrived at a 47% prevalence rate [55], providing further confirmation that the rates for Denmark and the Netherlands may be reliable (i.e. an additional 17.5% of flourishers in Denmark when using the MHC-SF as compared to Huppert and So’s operationalization).

Some considerations may be made regarding the flourishing rates reported in this study. Given that flourishing is conceptualized as maximized well-being located at the upper end of a well-being spectrum, we would expect much lower prevalence rates. However, the majority of the population in both Canada and Denmark are characterized by flourishing. Theoretically, we would expect the majority of the population to have moderate mental health [56], but in this case, it seems that the criteria for flourishing are too loose, consequently conflating flourishing with moderate mental health. If flourishing becomes the normal state of well-being in a population (pertaining to general characteristics of the majority), it seems the concept of flourishing is at risk of losing its meaning. In a previous study, we used more conservative criteria to operationalize flourishing, thereby capturing a population minority rather than a majority [55]. More conservative criteria for the operationalization of flourishing may also be warranted in the application of the MHC-SF. Another possibility might be that the criteria for flourishing should be determined for each country rather than an operationalization that assumes universal applications. That said, considering that there is an enormous amount of variation in MHC-SF flourishing rates between cultural settings, it is possible that the problem is inherently with the methodology used to operationalize flourishing within the MHC-SF, which may not be resolved simply by changing the criteria.

Some limitations of this study deserve mentioning. The response rate for the Danish survey was 34%, and while this is not unusual for web-based surveys [57], we cannot rule out that some degree of selection bias might have been introduced. Due to data encryption, we were not able to separate those with primary education from those with unknown education, which could have affected measurement invariance results. In terms of the cross-cultural comparison, there were differences in survey design in the different settings (web-based survey vs telephone and computer-assisted face-to-face interviews), and we were not able to test for measurement invariance across cultural settings (due to issues with data ownership), meaning that we cannot say with any certainty that the differences in well-being between the three countries are real differences or differences due to: a) problems with the scale, b) the scale performing differently in each cultural setting, and c) the surveys having different response rates and having been carried out in different ways, including minor differences in regards to the MHC-SF questionnaire and the way it was presented/answered.

## Conclusion

The bifactor modelling allowed us to observe that the scores for the MHC-SF are suitable for a comprehensive measuring of general well-being. This measure includes sources of variance of several substantive or theoretical dimensions – emotional, social, and psychological – which is consistent with an integral notion of positive mental health. Nonetheless, the scores are not suitable for using the subscales separately. This is consistent with the issues that arose in the qualitative study, particularly regarding the social dimension. The detailed analysis of the bifactor model showed that such issues pose no serious risks of bias for the general scores of well-being in the Danish MHC-SF data. The operationalization of flourishing or the criteria for it might also need a revision given that our cross-cultural comparison shows substantial variation in flourishing rates between settings, and for some countries much higher prevalence rates than could be expected from theory. However, these results should be seen in the light of the limitations reported, and more robust evidence is needed to determine the extent of revision needed. In particular, multi-country research (applying identical response categories) is warranted to test the construct validity (including measurement invariance testing) of the MHC-SF across national settings to be able to compare means across different cultural settings. In conclusion, the nature of the variance above the general factor that each subscale presented – particularly emotional and social– as well as the validity of the scale across cultural settings, deserve further study in future research.

## Supplementary information

**Additional file 1.**

## Data Availability

We do not have permission to share data.

## Abbreviations

MHC-SF: Mental Health Continuum - Short Form
PHQ4: The Patient Health Questionnaire for Depression and Anxiety (4-item version)
PSS: Perceived Stress Scale
