# Supplementary material for: Measuring positive mental health and flourishing in Denmark: validation of the mental health continuum-short form (MHC-SF) and cross-cultural comparison across three countries
Source: Health Qual Life Outcomes. 2020 Sep 4;18:297. doi: 10.1186/s12955-020-01546-2 (PMC7650216; doi:10.1186/s12955-020-01546-2)
Supplement: Supplementary file 1 — Additional file 1. [file 12955_2020_1546_MOESM1_ESM.docx]

**Appendix**

Data sources, designs, and samples.

**Canada:**

The Canadian Community Health Survey – Annual Component (CCHS) is a cross-sectional survey that collects information related to health status, health care utilization and health determinants for the Canadian population. It surveys a large sample of respondents and is designed to provide reliable estimates at the health region level. The CCHS data is collected from the population aged 12 years and older who live in private dwellings in over 100 health regions covering all provinces and territories in Canada. Excluded from the sampling frame are individuals living on Indian Reserves and on Crown Lands, institutional residents, full-time members of the Canadian Forces, youth aged 12 to 17 living in foster homes, and residents of certain remote regions. The CCHS uses two sampling frames for its sample selection: an area frame for the Canadian population aged 18 and over, and a list from the Canada Child Tax Benefit (CCTB) records for the population aged 12-17. The CCHS covers approximately 98% of the Canadian population aged 12 and over. The Mental Health Continuum – Short Form (MHC-SF) was implemented in the 2011, 2012 and 2015 Annual cycles of the CCHS. Analyses were conducted using data from the 2015 CCHS. In order to produce estimates that are representative of the national population, survey weights were incorporated in our calculations.

Reference:

Statistics Canada. Canadian Community Health Survey – Annual Component (CCHS) 2015 Share File. Ottawa, ON: Statistics Canada; 2017.

**The Netherlands:**

The Netherlands Mental Health Survey and Incidence Study-2 (NEMESIS-2) is a representative longitudinal cohort study that collects information related to diagnostic psychological disorders, mental health determinants and health care utilization. In this study, adults aged 18-64 at baseline were interviewed face-to-face in four waves (T0 = 2007-2009; T1 = 2010-2012; T2 = 2013-2015, T3 = 2016-2018). At baseline, respondents were recruited through a multistage, stratified, random sampling procedure of households, of which one respondent per household was randomly selected. A medical ethics committee (the Medical Ethics Review Committee for Institutions on Mental Health Care, METIGG) approved the study. After having been informed about the study aims, respondents provided written informed consent at each wave. A more comprehensive description of the design is provided in De Graaf et al. (2010). The mental health continuum-short form was implemented in 2010-2012 and 2013-2015. Analyses were conducted using data from the 2013-2015 wave. The data were weighted to correct for differences in response rates in several sociodemographic groups and in probability of selection of respondents within households at baseline.

Note: In the Dutch version of the MHC-SF, the response categories differ slightly from the original scale. This was done to make it easier for the respondents to recall. The answer categories are never, rarely, sometimes, regularly, often, or (almost) always.

Reference:

de Graaf, R., ten Have, M., van Dorsselaer, S., 2010. The Netherlands Mental Health Survey and Incidence Study-2 (NEMESIS-2): design and methods. Int. J. Methods Psychiatr. Res. 19, 125-141.
